# Survival of single-tooth implants in sites with and without history of external cervical root resorption: a register-based cohort study

**DOI:** 10.1186/s12903-026-08974-4

**Published:** 2026-06-20

**Authors:** Fernando José Mota de Almeida, Jonas Norberg Cedering, Mattias Pettersson

**Affiliations:** 1 Department of Endodontology, Institute of Odontology, The Sahlgrenska Academy, University of Gothenburg, Medicinaregatan 12 D, Gothenburg, 413 90 Sweden; 2 Department of Endodontics, Tandvårdens Kompetenscentrum, Public Dental Service, BOX 922, Skeppsbrogatan 22, Luleå, Region Norrbotten 971 28 Sweden; 3 Division of Prosthodontics, Department of Odontology, Umeå University, Norrlands Universitetssjukhus, 1D våning 8, Umeå, Västerbottens läns landsting 901 85 Sweden

**Keywords:** Dental implants, Implant survival, External cervical root resorption (ECRR), Registry-based cohort study, Kaplan–Meier analysis, Cox proportional hazards model

## Abstract

**Purpose:**

To compare the survival of single-tooth implants placed in sites with previous external cervical root resorption (ECRR) to those placed in unaffected sites.

**Methods:**

A retrospective matched cohort study was conducted using data from the Swedish National Dental Health Register (2009–2022). Patients receiving a single-tooth implant in a site with previous ECRR were identified and matched 1:1 to controls without such history by age, sex, tooth position, and timing of implant placement (± 2 years). Implant survival, defined as time from placement to removal, was analyzed using Kaplan–Meier curves and stratified Cox proportional hazards models. Restricted mean survival time (RMST) was estimated up to 10 years. Subgroup and sensitivity analyses assessed robustness.

**Results:**

The final cohort included 605 matched pairs (*n =* 1,210). The mean age was 52 years, and 56% of implants were placed in the maxillary anterior region. Implant extraction occurred in 12/571 (2.1%) sites with previous ECRR and 21/564 (3.7%) control sites. Ten-year cumulative survival was 97.3% for sites with previous ECRR and 94.4% for controls. The difference was not statistically significant (*HR* = 0.58; 95% *CI*: 0.28–1.22; *p* = 0.15). RMST analysis showed a negligible absolute survival difference (0.11 years; 95% *CI*: −0.04 to 0.25; *p* = 0.15).

**Conclusions:**

Within the limitations of registry-based data and a low number of failures, no statistically significant difference in implant survival was detected between sites with and without previous ECRR. These findings suggest comparable survival outcomes but should be interpreted with caution.

**Supplementary Information:**

The online version contains supplementary material available at 10.1186/s12903-026-08974-4.

## Background

External cervical root resorption (ECRR) is a pathological loss of tooth structure caused by clastic cell activity, occurring at the cervical region of the tooth just below the epithelial attachment. It is initiated by destruction of the cementum, which allows clastic cells from the periodontium to interact with dentine. The condition often begins asymptomatically, may progress through resorptive and reparative phases, and can eventually extend into the pulp space in advanced cases. The clinical presentation is variable, depending on the location, severity, and nature of the lesion [1–3]. Although ECRR usually affects a single tooth, multiple teeth may occasionally be involved [4, 5]. The prevalence of ECRR has been reported to range from 0.6% to 8% in studies based on cone-beam computed tomography (CBCT) imaging, although these estimates may be influenced by sampling bias [6–8]. In contrast, a Danish cross-sectional study of adolescents, mean age 17.8 years, reported a CBCT-verified prevalence of approximately 0.18% [9].

Several factors have been associated with ECRR, including orthodontic treatment (commonly reported), dental trauma, parafunctional habits, intra-coronal bleaching following trauma, and surgical or operative trauma such as periodontal treatments. However, most supporting evidence comes from observational studies with limited control [10, 11]. In a case-control study, diabetes was the only systemic factor, and dental trauma the only local factor, significantly associated with ECRR [12]. Treatment options vary depending on lesion severity, location, and aetiology. Management of ECRR aims to remove the resorptive tissue, repair the cavitated defect with a suitable restorative material, and monitor the affected tooth for recurrence. The feasibility and prognosis depend on the extent, location, and accessibility of the lesion [3]. When lesions are non-restorable or conservative treatments fail, extraction is often necessary. Because most lesions are asymptomatic, they are often detected at advanced stages, making extraction the only viable option. Even conservative management of minor lesions can fail, leading to eventual tooth loss; in one study, 33% of teeth were extracted as the initial treatment, and a further 11% within 10 years despite attempts at preservation [13].

Dental implants are widely regarded as the standard treatment for replacing missing teeth, from single tooth loss to complete edentulism [14]. Millions of implants are placed worldwide annually, demonstrating predictable functional and aesthetic outcomes [15, 16]. Despite high survival rates, implant therapy is not without complications. Biological issues such as peri-implant mucositis and peri-implantitis, as well as technical problems including abutment or screw loosening, loss of retention, and veneering materials fractures, have been reported in long-term studies [17, 18]. Furthermore, risk factors such as smoking, history of periodontitis, and poor bone quality may further compromise outcomes [19, 20].

ECRR involves pathological clastic activity and localized inflammatory processes affecting mineralized dental tissues. Whether these biological characteristics are associated with host-related factors that could influence peri-implant bone remodelling or long-term implant survival remains unclear, prompting questions regarding implant outcomes in sites previously affected by ECRR. Given the increasing use of implants and the potential for ECRR-related tooth loss, understanding outcomes in these cases is essential for evidence-based treatment planning [21, 22]. Evidence on the survival of implants placed in sites where teeth were extracted due to ECRR is scarce. There are case reports describing successful implant therapy in such scenarios; however, these reports tend to emphasize positive outcomes [23]. While osseointegrated implants are considered a predictable treatment for single-tooth replacement, with long-term survival rates often exceeding 90% over 10–20 years, most data derive from teeth lost for reasons other than ECRR [24, 25]. It remains unclear whether a history of ECRR at the extraction site influences implant survival outcomes. To date, only isolated case reports have documented successful implant therapy following extraction for ECRR, highlighting the need for robust data to inform clinical decision-making [26–29].

In Sweden, the national dental insurance system administered by Swedish Social Insurance Agency (SSIA) enables longitudinal tracking of dental procedures and diagnoses, including ECRR-related treatments. This register allows for large-scale analyses and helps overcome one of the main limitations of previous studies—small sample sizes of consecutive patients— at the expense of detailed clinical characterization.

## Methods

### Aim

The primary aim is to compare the survival of single-tooth implants placed in sites with previous ECRR to those placed in sites without such history. Implant survival will be evaluated across the entire observation period, including early (prior to loading) and late (post-loading) failures.

It was hypothesized that there is no difference in the survival of single-tooth implants between sites with a previous ECRR and those without.

### Study design and settings

This retrospective, nationwide, registry-based cohort study was conducted in Sweden and adhered to the STROBE guidelines for observational studies [30]. Data were obtained from the National Dental Health Register maintained by the National Board of Health and Welfare, using information provided by the SSIA. The SSIA register is reimbursement-driven and includes all adult patients receiving subsidized dental care (https://www.forsakringskassan.se/english/dental-care-subsidy). A validation study has demonstrated high accuracy for key variables, including a 91.5% positive predictive value for the number of remaining teeth [31]. Study size was determined by the available nationwide registry population for the ECRR-group, reflecting the total number of eligible cases identified during the study period.

Register and coding changes during the study period (e.g., introduction of first-molar implant codes in 2014) were documented and addressed during data cleaning (see Supplementary Table S1).

### Participants and eligibility criteria

The study group included all patients in the register (> 19 years old) who received a single-tooth implant between 2009 and 2023 in a site where the extracted tooth had previously been assigned a diagnosis of ECRR between 2009 and 2018 (hereafter referred to as ‘sites with previous ECRR’), regardless of whether the diagnosis was registered at the time of extraction (codes listed in Supplementary Table S1). If a patient had multiple implants, only the first eligible implant was included to ensure the independence of observations and avoiding clustering effects.

Controls were selected and matched 1:1 to cases by age, sex, tooth position (contralateral sites accepted), and timing of implant placement (within 1–2 years). Matching was performed using a deterministic algorithm implemented by the registry team, which prioritized exact matches on sex and tooth position, followed by nearest-neighbour matching on age and year of implant placement within the specified tolerance (± 2 years). Only one implant per individual was included using the same criterium as for the study group. All data were anonymized.

### Data source and variables

Variables extracted for this study included patient demographics (age, sex), tooth position, diagnostic codes (including ECRR), treatment codes for implant placement and removal, implant-supported crown placement, pre-implant adjunctive procedures such as bone augmentation and oral health. The final matched cohort comprised 605 pairs (*n =* 1,210) and was used for all analyses except oral health variables. The registry data set contained no missing values for variables required for cohort definition, matching, exposure classification, or outcome assessment. Records with implausible values for oral health variables (0 remaining teeth or 32 intact teeth) were excluded in accordance with published validation criteria [31], no data imputation or replacement was performed. This resulted in analytic sample sizes of 577 individuals in the ECRR group and 557 in the control group (total *n* = 1,134) for analyses involving oral health measures.

### Outcome definitions

The primary outcome was implant survival, defined as the period the implant remained in situ until removal due to failure. Implants that were removed were considered failures regardless of whether they had received a prosthetic restoration. Implants that never had any registered restoration (hereafter labelled as unloaded) and for which no other treatment was recorded were generally excluded from the primary survival analysis, but all unloaded implants were analysed separately to assess potential differences between study groups. Unloaded implants that were known to have been removed were counted as failures. Loaded implants that were subsequently removed were classified as failures, whereas implants that remained in situ at the end of follow-up were censored. When no explicit removal record was present, proxy indicators were used to infer implant failure, such as subsequent placement of a new implant in the same position, registration of a pontic, or a removable prosthesis in that position either following an implant-supported crown or without any record of a subsequent implant-supported crown.

This classification was manually validated by cross-referencing treatment sequences and tooth positions over time using only the information available in the SSIA register. Individuals with inconsistent or implausible treatment registrations that could not be reliably interpreted were excluded from the analysis, along with their matched pairs (e.g., removal codes preceding implant placement or unrealistically short intervals between consecutive implants). The validation was performed independently by all three investigators (FMA, JNC, MP), and any discrepancies were resolved by consensus. While registry-based definitions have inherent limitations, these criteria provide a consistent and reproducible approach for comparative analysis between groups.

### Observation period and censoring

The observation period covered implant placements from January 1, 2009, to December 31, 2022. Baseline for the primary survival analysis was defined as the date of implant placement. Implants were followed until a terminal event (implant removal) or censoring at emigration, death, or December 31, 2023.

### Statistical analysis

*Null hypothesis* (H_*0*_): there is no difference in the survival of single-tooth implants between sites with previous ECRR and sites without previous ECRR.

Descriptive statistics were used to summarize baseline characteristics. Differences between matched pairs were assessed using McNemar’s test for dichotomous variables, Cohen’s Kappa for categorical variables, and paired t-tests or Wilcoxon signed-rank tests for continuous variables.

Differences between the matched groups in the proportion of unloaded implants were analysed using McNemar’s test. Implant survival was analysed using Kaplan–Meier curves generated using R/*survminer*. Group comparisons were performed with stratified Cox proportional hazards models to account for matching. Univariate Cox regression models were stratified by matched pairs to account for the matching design. In addition to hazard ratios, restricted mean survival time (RMST) up to 3,653 days (~ 10 years) and restricted mean time lost (RMTL) were estimated to provide an absolute measure of survival differences between groups.

Subgroup analyses were pre-specified for sex, age, tooth position, bone augmentation, and number of remaining and intact teeth. Additional sensitivity and robustness analyses were performed (including multistate transition modelling); details are provided in the Supplementary Materials. Analyses were performed using IBM SPSS Statistics for Windows, Version 29.0.1.0 (IBM Corp., Armonk, NY, USA) and R (version 4.5.0). All tests were two-sided, and a p-value < 0.05 was considered statistically significant. Confidence intervals were calculated at the 95% level.

## Results

A total of 605 individuals were included in the study group and matched to 605 controls, after exclusion of 9 individuals (and their matched pairs) due to inconsistencies in the register. Matching quality was high, with perfect or near-perfect concordance for sex and tooth position (Kappa = 1.000 and 0.997, respectively). Minor differences in age and year of implant placement were statistically significant due to the large sample size but clinically negligible (mean difference < 0.5 years). For implants with available tooth extraction dates across both groups (*n* = 758), the interval from tooth extraction to implant placement showed a highly right-skewed distribution, with a mean of 314 days (*SD* 559; range 0–4.754). Comparable data were not consistently available for the remaining controls due to registry-matching constraints.

**Fig. 1:**
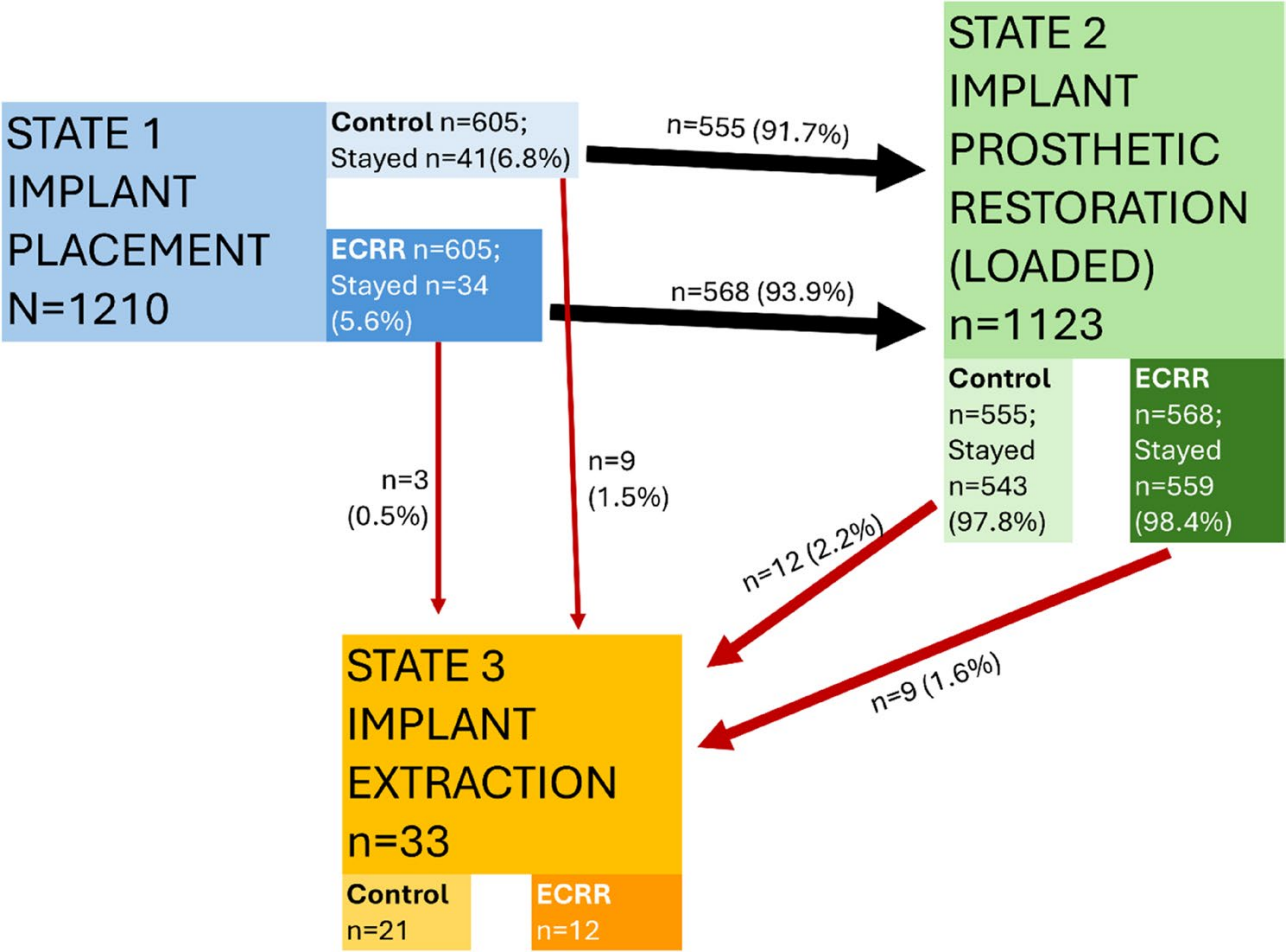
State-transition diagram illustrating the implant progression from placement (State 1) to restoration (State 2) or extraction/failure (State3). Most implants reached the loaded state (State 2), and very few transitioned directly to extraction (State 3). Numbers within boxes indicate total implants per state, stratified by group (ECRR and control). Detailed transition probabilities and analytical methods are provided in the Supplementary Material

The majority of implant sites was in the maxillary anterior region (56.2%). Bone augmentation was uncommon and evenly distributed (0.3% in both groups). Baseline characteristics and group comparisons are presented in Table 1. The ECRR group had a significantly higher number of remaining and intact teeth compared to controls (*p* < 0.001), indicating better baseline oral health. Figure 1 illustrates the transitions between implant placement (State 1), successful prosthetic restoration (healthy State 2), and implant extraction (failure State 3).

**Table 1:** Baseline characteristics of ECRR and control groups

Characteristic	ECRR Group (*n* = 605)	Control Group (*n* = 605)
Demographics		
* Age* (years)	52.27 (15.99)^**†**^	52.67 (15.92)^**†**^
* Sex*		
Male	316 (52.2%)	316 (52.2%)
Female	289 (47.8%)	289 (47.8%)
Implant Characteristics		
* Tooth Position*		
Maxillary front	340 (56.2%)	340 (56.2%)
Maxillary premolars	53 (8.8%)	54 (8.9%)
Maxillary molars	38 (6.3%)	37 (6.1%)
Mandibular front	47 (7.8%)	47 (7.8%)
Mandibular premolars	64 (10.6%)	64 (10.6%)
Mandibular molars	63 (10.4%)	63 (10.4%)
* Year of placement*	8.10 (2.62)^**†**^	8.16 (2.66)^**†**^
* Bone augmentation*	4 (0.3%)	4 (0.3%)
Oral Health		
* Number of remaining teeth*	27.76 (3.10)^**†**^	25.88 (5.13)^**†**^
* Number of intact teeth*	16.05 (8.27)^**†**^	14.39 (9.17)^**†**^
* Excluded information on oral health*	28 (4.63%)^**‡**^	48 (7.93%)^**‡**^

Values are presented as *n* (%) or mean (*SD*)^†^. Values are based on the full matched cohort (605 per group; total *n* = 1,210). ^‡^For oral health variables, analytic sample sizes were lower due to exclusion of records showing implausible values (0 remaining teeth or 32 intact teeth) based on a previous validation study [31]. Matching quality was assessed using McNemar’s test (dichotomous), Cohen’s Kappa (categorical), and paired t-tests or Wilcoxon signed-rank tests (continuous). Detailed statistical test results are provided in the text

Unloaded implants were observed in 37/605 implants in the ECRR group (6.1%; 95% *CI*: 4.5–8.3) and 50/605 implants in the control group (8.3%; 95% *CI*: 6.3–10.7); this difference was not statistically significant (McNemar test, two-sided, *p* = 0.177).

**Fig. 2:**
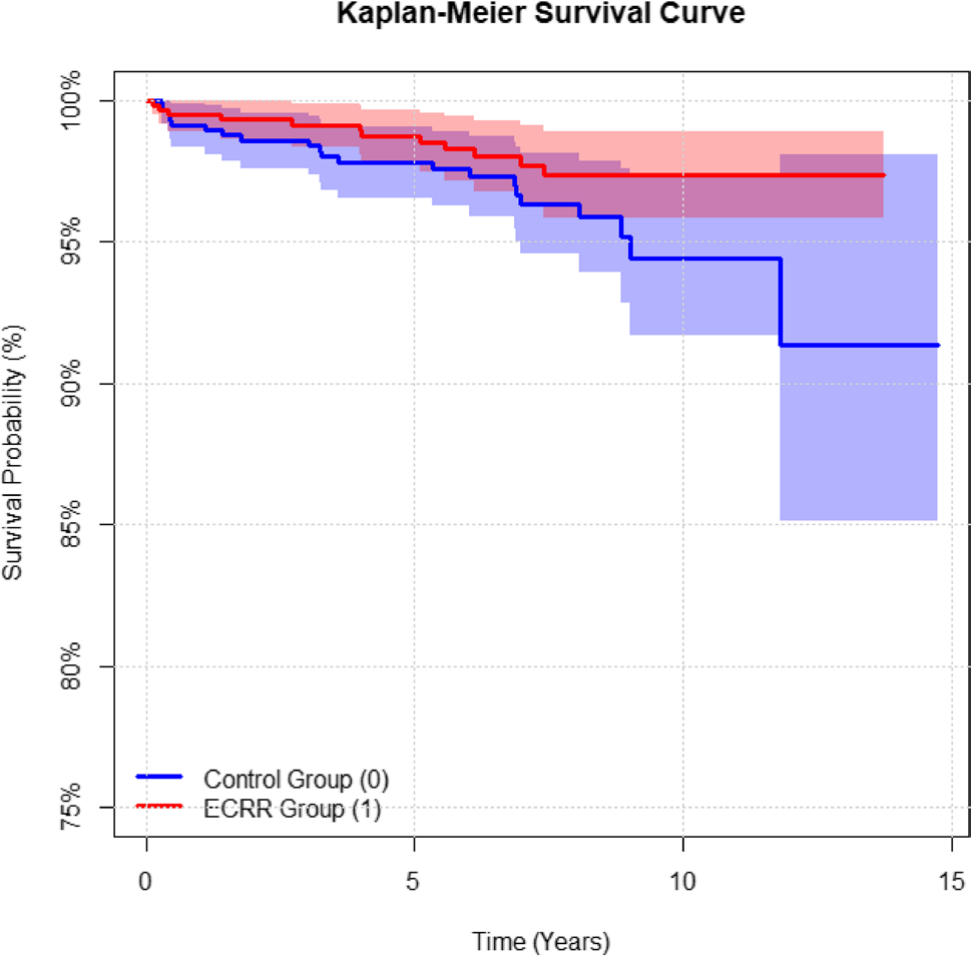
Kaplan–Meier survival analysis of implant survival over the entire observation period. The curves illustrate cumulative survival probability for two groups: the Control group (blue) and the ECRR group (red). The x-axis shows time in years (0–15), and the y-axis shows survival probability (75% – 100%). Shaded areas indicate 95% confidence intervals. *p* > 0.05 between groups

Implant extractions occurred in 12/571 implants (2.1%; 95% *CI*: 1.2–3.6) in the ECRR group and 21/564 implants (3.7%; 95% *CI*: 2.4–5.6) in the control group. Of the total number of implant failures (*n* = 33), 17 (51.5%) had a directly registered extraction cause—most of which were biological failures (*n* = 14)—while the remaining 16 (48.5%) were identified through predefined proxy indicators. These proportions were similar across groups, with no indication of imbalance between direct and proxy-defined events. No meaningful imbalance between groups was observed in the proportion of direct versus proxy-defined events, although the overall number of terminal events was small. The mean observation time for individuals without extraction events was 7.1 years (*SD* 2.73; range 0.1–14.7). Kaplan–Meier analysis showed similar survival patterns for both groups (Fig. 2). The cumulative survival at one year was 99.5% for the ECRR group and 99.1% for controls. At 10 years, survival remained high in both groups (97.3% vs. 94.4%). Complete annual survival data are presented in Table 2.

**Table 2:** Kaplan–Meier cumulative survival by year over a 10 year-period

Year	At risk		Cumulative events		Survival % (95% CI)	
	ECRR	Control	ECRR	Control	ECRR	Control
*0*	571	564	0	0	100.0 (100.0–100.0)	100.0 (100.0–100.0)
*1*	568	548	3	5	99.5 (98.9–100.0)	99.1 (98.3–99.9)
*2*	552	536	4	8	99.3 (98.7–99.9)	98.6 (97.6–99.6)
*3*	531	514	5	8	99.1 (98.3–99.9)	98.6 (97.4–99.8)
*4*	505	480	6	12	98.9 (98.1–99.7)	97.8 (96.6–99.0)
*5*	461	433	7	12	98.7 (97.7–99.7)	97.8 (96.6–99.0)
*6*	378	361	9	13	98.3 (97.1–99.5)	97.5 (96.1–98.9)
*7*	315	293	11	17	97.7 (96.3–99.1)	96.3 (94.5–98.1)
*8*	220	216	12	17	97.3 (95.7–98.9)	96.3 (94.5–98.1)
*9*	150	125	12	19	97.3 (95.7–98.9)	95.2 (92.8–97.6)
*10*	63	55	12	20	97.3 (95.7–98.9)	94.4 (91.7–97.1)

In a stratified Cox regression analysis, the risk of implant extraction was not significantly different between groups (hazard ratio [*HR*] = 0.58, 95% *CI*: 0.28–1.22; *p* = 0.15). Matching variables (sex, tooth position) were constant with strata and thus not estimated separately. Other variables, including age, bone augmentation and number of remaining teeth, did not significantly affect outcomes (*p* > 0.05). RMST analysis up to 10 years showed minimal differences between groups: 9.85 years for ECRR versus 9.74 years for controls (difference 0.11 years; 95% *CI*: −0.04 to 0.25; *p* = 0.15), consistent with Kaplan–Meier and Cox regression findings. None of the additional sensitivity and robustness analyses indicated a statistically significant difference with clinical relevance between groups (see Supplementary Materials).

## Discussion

This nationwide cohort study found that single-tooth implants placed in sites with a history of ECRR exhibited long-term survival similar to implants in unaffected sites, with no statistically significant difference detected between groups. At 10 years, cumulative survival was 97.3% for sites with previous ECRR and 94.4% for controls, suggesting that implant therapy appears to be a predictable treatment option even in these previously affected sites, although smaller differences between groups cannot be excluded.

These findings can be contextualized within the broader literature on single-tooth implant survival. Long-term studies consistently report survival rates exceeding 95% over 10–20 years, with early failures being uncommon and most implants progressing to a loaded state [24, 32]. Our results align with these observations, showing excellent survival and minimal early losses, reinforcing the durability of implant therapy. Multiple statistical approaches consistently demonstrated similar implant performance after ECRR and in controls. Based on the observed event rate and sample size, the study had approximately 80% power to detect hazard ratios of ≥ 2.65, but substantially lower power for smaller effect sizes (e.g., 51% for HR = 2.0 and 21% for HR = 1.5). Therefore, while large differences can be excluded with reasonable confidence, smaller but potentially clinically relevant differences between groups may not have been detected (see Supplementary Materials, Sect. 4.4). Sensitivity analyses and triangulation across methods further support the robustness of these findings.

Evidence on implants placed after ECRR-related tooth loss has been limited to case reports and one small observational cohort, which provide insufficient data for meaningful comparison [23, 26, 33, 34]. By leveraging a large nationwide registry and matched design, this study addresses that gap and offers robust estimates of implant survival in this specific clinical scenario.

Interpretation of baseline differences provides additional insights. The slightly lower survival in the control group may reflect differences in baseline oral health, as controls had fewer remaining and intact teeth, potentially indicating a higher prevalence of periodontal disease—a known risk factor for implant complications [35]. Adjustment for remaining and intact teeth in the stratified Cox sensitivity analyses did not materially change the hazard ratio or alter the interpretation of the group comparison, although the low number of extraction events limits the strength of these adjusted estimates. The mean age in our cohort was approximately 52 years, which is higher than in most long-term single-tooth implant follow-up studies, where patients are often in their 20–30 s at the time of treatment [32]. This difference might reflect that ECRR-related tooth loss occurs later in life compared to loss caused by trauma or maxillary lateral incisors agenesis that typically affects adolescents and young adults [36, 37]. Bone augmentation was uncommon (0.3%) and evenly distributed between the groups, indicating that ECRR-related tooth loss rarely necessitated additional bone grafting prior to implant surgery, likely because the resorptive process is localized and does not result in major alveolar bone loss. Failure timing was evaluated using Kaplan–Meier curves, cumulative survival tables, and multistate modelling and did not differ among groups. Analysing unloaded implants separately allowed us to evaluate early healing behaviour without assuming that non-restoration represented implant failure, and no differences between groups were detected.

Strengths of this study include the large sample size, long follow-up, and high-quality registry data, enabling an analysis that would otherwise be difficult due to the low prevalence of ECRR.

A major limitation is that the reliability and validity of our findings depend on accuracy of registry coding and reporting. To our knowledge, no formal validation study of the ECRR diagnostic code within the SSIA register currently exists. Therefore, the ability of this code to accurately identify true clinical ECRR cases cannot be confirmed with certainty, and some degree of misclassification must be assumed. However, the extremely small number of registered cases—far below 0.02% of annual SSIA submissions—suggests that miscoding with more common diagnoses (e.g., caries) is unlikely. Our interpretation also presumes that the dentists who do use this code are those familiar with the condition. The SSIA resorption code describes defects treatable with conservative restorations and closely matches the clinical presentation of ECRR, supporting high specificity. Underreporting is more likely, for example when ECRR is diagnosed as another condition. However, this would mainly reduce sample size without biasing comparisons between the matched groups. Furthermore, only Swedish dentists can report professionally to this register, a reimbursement system with oversight, reducing misreporting. A recent audit of a randomized sample of the register showed that 94% of the co-payment volume was correctly assigned, indirectly suggesting high overall data accuracy of the register [38]. Furthermore, analyses using the same registry to evaluate tooth survival following endodontic treatment have produced findings consistent with those from smaller clinical observational studies. This is notable even though the underlying diagnostic and treatment codes were not formally validated, and indirectly supporting the reliability of the register [39–41]. Reporting accuracy is further supported by earlier validation studies of oral-health indicators within the same register [31]. Another major limitation is the absence of detailed clinical peri-implant data—such as bone level changes, probing depths, peri-implant mucositis/peri-implantitis, smoking status, surgical technique, and loading protocols—an inherent constraint of registry-based studies that restricts interpretation of biological outcomes beyond implant survival. Periodontitis diagnoses were not included due to non-specific coding and high prevalence related to routine periodontal care; however, residual confounding from periodontal status cannot be fully excluded, although adjustment using the number of intact teeth partly addresses this limitation. Peri-implantitis is subject to similar limitations, as diagnoses are not consistently recorded in a site-specific manner and are severely under-reported in the register. Consequently, peri-implant disease cannot be reliably identified or interpreted, and peri-implantitis and its management were therefore not analysed. Accordingly, the low number of recorded peri-implant complications in this study should not be interpreted as reflecting true clinical incidence, but rather as a known limitation of registry-based data. Immediate implant placement could be identified in some cases (*n* = 78), but immediate loading could not. Tooth extraction–implant intervals could not be analysed comparatively because extraction dates were unavailable for a substantial proportion of control sites; therefore, any potential influence of placement timing on outcomes cannot be ruled out. The proxy definitions are systematic and reproducible, but reliance on indirect indicators for a subset of failures is a meaningful limitation that may reduce the precision of event classification, even though no systematic differences between groups were evident. In summary, the main limitations of this study relate to (1) uncertainty in diagnostic coding, (2) partial reliance on proxy definitions of implant failure, and (3) lack of detailed clinical peri-implant variables and other potentially important confounders, such as smoking status and systemic conditions, inherent to registry-based research. Despite these constraints, registry-based studies provide a broad, real-world perspective on population-level outcomes, albeit with limited clinical detail. These limitations are unlikely to differ systematically between matched groups or materially affect the comparative findings. Albeit, they may influence the interpretation and generalizability of the overall survival estimates. The calculated E-value indicates that only a moderately strong unmeasured confounder could explain the observed associations, suggesting residual confounding is unlikely to materially affect conclusions.

Given that most implants were placed in the anterior maxilla, predictable outcomes in this region are critical for aesthetics and patient confidence. These findings are clinically relevant and may provide reassurance to both patients and clinicians considering implant therapy in sites previously affected by ECRR. Beyond direct clinical implications, the study illustrates the value of registry-based research for rare conditions, enabling population-level analyses that overcome the limitations of small clinical cohorts and allow linkage with other health data for interdisciplinary research. Such approaches can inform healthcare policy, resource allocation, and evidence-based planning, while the comprehensive, population-level nature of the registry data supports high external validity in similarly well-developed healthcare systems. Validation through prospective multi-center studies with detailed peri-implant health data would further strengthen these conclusions, and additional confirmation through pooled analyses or extended follow-up could enhance generalizability. Finally, the effect estimates reported here can guide hypothesis generation and power calculations for future controlled studies.

## Conclusions

Single tooth implants placed in sites with a history of ECRR showed survival rates comparable to those in unaffected sites. Within the limitations of registry-based diagnosis—particularly the lack of condition specific validation and the small number of failures—no statistically significant difference in implant survival was detected within the limits of the available sample size and event rate. These findings should be interpreted with caution, and confirmation through prospective studies with validated diagnostic criteria and detailed peri-implant assessments is needed. Nonetheless, this study currently provides the most comprehensive evidence available on implant survival following ECRR related tooth loss.

## Supplementary Information

Supplementary Material 1.

## Data Availability

The datasets used and/or analysed during the current study are available from the corresponding author on reasonable request.

## Abbreviations

ECRR: External cervical root resorption
RMST: Restricted mean survival time
SSIA: Swedish Social Insurance Agency
