# Supplementary material for: Survival of single-tooth implants in sites with and without history of external cervical root resorption: a register-based cohort study
Source: BMC Oral Health. 2026 Jun 20;26:1088. doi: 10.1186/s12903-026-08974-4 (PMC13282867; doi:10.1186/s12903-026-08974-4)
Supplement: Supplementary file 1 — Supplementary Material 1. [file 12903_2026_8974_MOESM1_ESM.docx]

## **Supplementary material**

1. Registry coding and policy changes

Full details of code definitions and updates are available at <https://husk.tlv.se/> (in Swedish). During the observation period, the Swedish National Dental Health Register underwent updates that may influence data interpretation and coding consistency. The study harmonized codes across years to ensure comparability.

- **2014:** Insurance coverage for implants in the position of the first molars was introduced; coding and reimbursement rules were updated accordingly.
- **2017:** The upper age limit for free dental care was extended from 19 to 23 years, temporarily excluding individuals aged 19–23 from the register.
- **Additional refinements:** Selected codes were discontinued or introduced and treatment categories adjusted to align with evolving clinical practice and reimbursement policies.

All changes were documented and accounted for during data cleaning. Analyses were restricted to codes consistently available throughout the study period.

2. Coding transparency and National Dental Health Register code list (Supplementary Table S1)

A complete list of diagnostic and treatment codes used in this study (including those for external cervical root resorption (ECRR), implant placement, implant removal, prosthetic restorations, bone augmentation, and proxy indicators for implant failure) is provided below as Supplementary Table S1. The table also notes any code changes and their effective dates when relevant.

Table S1. Complete list of diagnostic and treatment codes with descriptions and notes

| **Category** | **Code** | **Description** | **Notes** |
| --- | --- | --- | --- |
| *Diagnosis* | 4074 | Cavity due to external resorption | Permits only filling therapies or surgical procedures for fillings/extractions; implies ECRR since other external resorptions do not match the permitted treatments. |
| *Implant placement* | 420 | Implant, per unit, additional procedure |  |
|  | 421 | Surgery concerning a jawbone-anchored implant, one implant | Introduction of implant codes for first molars in 2014. |
|  | 925 | Replacement procedure: implant in a single-tooth gap instead of a tooth-supported bridge, surgical part / implant surgery | Introduction of implant codes for first molars in 2014. |
| *Implant removal* | 429 | Surgical removal of an implant |  |
|  | 435 | Removal of an implant | Discontinued in 2022. |
|  | 436 | Removal of an implant | Prior to 2022, recorded as “easy implant removal.” |
|  | 404 | Surgical removal of multiple teeth or other tissue in the same quadrant | Two cases (one per group) were considered true implant extractions. |
| *Prosthetic restorations* | 850 | Implant-supported crown, one per jaw |  |
|  | 852 | Implant-supported crown, several in same jaw | Used when placed simultaneously with others, whether single or part of a bridge. |
|  | 890 | Implant-supported crown on an existing implant |  |
|  | 926 | Replacement procedure: implant-supported crown (single-tooth gap instead of a tooth-supported bridge), prosthetic component |  |
|  | 929 | Replacement procedure: implant treatment in a single-tooth gap when tooth-supported crowns (800/801) were performed within three years on abutment teeth; includes prosthetic component |  |
| *Proxy indicators* | 420/421/925 | See implant placement codes | Used as a proxy for implant extraction only when an implant had been previously placed. |
|  | 804 | Pontic unit in tooth-supported prosthesis, per unit | Not a proxy if used as interim prior to implant restoration (e.g., resin-bonded bridge). Considered a proxy if no other replacement was ever registered or if recorded following an implant-supported crown. |
|  | 853 | Pontic unit in implant-supported prosthesis |  |
|  | 822–823 | Partial denture without metal framework / temporary (822: 1–3 teeth; 823: ≥4 teeth) | Same proxy criteria as code 804. |
|  | 824 | Partial denture with a metal framework, anchored using metal retainers | Same proxy criteria as code 804. |
|  | 827–829 | Complete denture (827: lower; 828: upper; 829: immediate) | Considered only when congruent with tooth position. |
| *Bone augmentation* | 427 | Bone grafting using autologous bone in a single quadrant |  |
|  | 428 | Bone augmentation with bone substitute material in one quadrant |  |
|  | 430–432 | Bone augmentation (autologous or substitute) in conjunction with another procedure; additional procedure |  |
|  | 433 | Sinus lift without autologous bone or bone substitute material in conjunction with implant placement, per quadrant; additional procedure |  |

3. Supplementary analyses (methods)

3.1 Baseline

All analyses were performed in R (version 4.5.0). Data manipulation and visualization were conducted with the *tidyverse* suite. Baseline covariate balance between the ECRR and control groups was evaluated using standardized mean differences (SMD), with |SMD| < 0.1 indicating adequate balance. Covariate balance was visualized in a Love plot generated with the *cobalt* and *ggplot2* packages (Figure S1). To account for within-patient and within–matched-pair correlations, two complementary approaches were applied: generalized estimating equations (GEE) with robust standard errors clustered by patient (*gee* package), and linear mixed-effects models with random intercepts for matched pairs and individual patients (*lme4* package). Statistical significance was defined as two-sided *p* < 0.05.

3.2 Unloaded implants

Univariable conditional logistic regression (*survival*::*clogit*) was used to explore associations between baseline variables and the likelihood of having an unloaded implant. A multivariable model including study group and the number of remaining teeth was then fitted to compare ECRR versus control while addressing the imbalance in remaining teeth (selected based on borderline association in univariable analyses).

3.3 Implant survival

A stratified Cox proportional hazards regression compared implant extraction between groups, adjusting for baseline covariates that remained unbalanced (number of remaining teeth and number of intact teeth), with matched pairs as strata. The proportional hazards assumption was assessed using Schoenfeld residuals (*cox.zph*), both numerically and visually. Residuals were plotted over follow-up time to inspect for systematic deviations from zero. Restricted mean survival time (RMST) up to 3,653 days (~10 years) and restricted mean time lost (RMTL) were estimated with *survRM2*::*rmst2*. A pre-specified sensitivity analysis evaluated robustness to alternative implant failure definitions: the primary definition combined explicit implant removal with proxy indicators (e.g., subsequent implant placement, pontics, or removable prostheses), whereas the strict definition considered only explicit removal. Kaplan–Meier (KM), RMST, and stratified Cox models were recalculated under both definitions.

3.4 Power and robustness to unmeasured confounding

A post hoc power analysis was performed using the Schoenfeld approximation formula implemented in base R to determine the minimum detectable hazard ratio (HR). E-values were then calculated manually using the formula *E = HR + √[HR × (HR − 1)]* (with reciprocal transformation applied for protective effects) to assess the robustness of observed associations to potential unmeasured confounding.

3.5 Multistate analyses

A multistate transition framework was used to examine longitudinal dynamics across three clinical states: State 1 (implant placement), State 2 (healthy condition / implant-supported crown placed), and State 3 (implant extraction, terminal event). Transitions were derived from observed sequences; person-time accrued from entry into each state until extraction or censoring. Kaplan–Meier estimators and log-rank tests were used for transition probabilities; Poisson methods yielded incidence rates (IR) and incidence rate ratios (IRR) with 95% *CI*; and Cox models estimated transition-specific hazard ratios. Kaplan–Meier curves with shaded 95% *CI*, cumulative incidence, and survival tables were generated to depict survival dynamics (Figures S2–S3; Tables S2–S3).

**4. Supplementary results**

**4.1 Baseline**

Most baseline covariates achieved adequate balance (|SMD| < 0.1). Residual imbalance persisted for the number of remaining and intact teeth (Figure S1), but the limited number of events and lack of association with outcomes did not justify post-matching adjustments. At the patient level, the ECRR group demonstrated significantly better baseline dental status, with a mean of 1.66 more intact teeth (95% *CI*: 0.88 – 2.43; *p* = 3.29 × 10⁻⁵) and 1.87 more remaining teeth (95% *CI*: 1.43 – 2.32; *p* = 1.11 × 10⁻¹⁵) than controls; findings were consistent across paired t-tests, generalized estimating equations, and mixed-effects models, confirming the robustness of this finding.

**4.2 Unloaded implants**

In matched-pair analyses, the odds of implant failure showed a non-significant trend towards lower odds in the ECRR group (OR 0.72, 95% *CI*: 0.46 – 1.12; *p* = 0.15). Age and number of intact teeth were not associated with the outcome (OR per year = 0.90, 95% *CI*: 0.68 – 1.19, *p* = 0.47; OR per tooth = 0.99, 95% *CI*: 0.94 – 1.05, *p* = 0.82). A borderline trend was observed for number of remaining teeth (OR per tooth = 0.93, 95% *CI*: 0.86 – 1.01, *p* = 0.068). In models adjusting for number of remaining teeth, no between-group difference was detected (OR 0.91, 95% *CI*: 0.55–1.52). Analyses of sex, implant position, and bone type were not interpretable due to sparse events or lack of discordant pairs within strata. Overall, no variable reached statistical significance.

**4.3 Implant survival**

After adjusting for remaining and intact teeth, the hazard ratio for extraction comparing ECRR versus control was 0.65 (95% *CI*: 0.25 – 1.70). An alternative model estimated HR = 0.55 (95% *CI*: 0.27 – 1.11; *p* = 0.09), consistent with RMST and Kaplan–Meier analyses. The proportional hazards assumption was not violated (global *p* = 0.45). RMST up to 10 years was 9.85 years (ECRR) versus 9.74 years (control); the difference was 0.11 years (95% *CI*: −0.04 – 0.25; *p* = 0.148). Sensitivity analyses using the strict failure definition yielded HR = 0.79 (95% *CI*: 0.29 – 2.09; *p* = 0.618) and nearly identical RMST values (difference −0.43 days; 95% *CI*: −21.38 – 20.52; *p* = 0.97).

**4.4 Power and robustness to unmeasured confounding**

The study had 80% power to detect HR ≥ 2.65, but only 21.3% for HR = 1.5 and 51.2% for HR = 2.0. E-values were 1.74 (unadjusted HR = 0.58) and 2.79 (adjusted HR = 0.65), indicating that moderately strong unmeasured confounding would be required to explain the observed associations

**4.5 Multistate analyses**

Transitions are summarized in Supplementary Table S2. From State 1, the ECRR group had fewer direct transitions to extraction and a very high proportion reaching State 2. Median time to State 2 was shorter for ECRR (119 days) than controls (129 days) (log-rank χ² = 5.5, *p* = 0.02; Figure S2). For State 2 → 3, no significant difference was observed (log-rank χ² = 0.004, *p* = 0.95; Figure S3). Extraction incidence was numerically lower in ECRR (IRR = 0.554, 95% *CI*: 0.248 – 1.179; *p* ≈ 0.12). Kaplan–Meier estimates are provided in Supplementary Table S3.

5. Supplementary Tables

**Supplementary Table S2**. State transition patterns by study group (actual transitions only)

| **From State → State / Status** | **Control**  **(n, %)** | **ECRR**  **(n, %)** | **Person-Years**  **(Control / ECRR)** | **IR**  **(per 1000 PY)** | **IRR**  **(ECRR vs Control)** |
| --- | --- | --- | --- | --- | --- |
| ● → ○ | 555 (91.7%) | 568 (93.9%) | 583 / 500.4 | – | – |
| ● → ✖ | 9 (1.5%) | 3 (0.5%) | 583 / 500.4 | 15.44 / 6.00 | 0.388 |
| ● Stayed | 41 (6.8%) | 34 (5.6%) | – | – | – |
| ○ → ✖ | 12 (2.2%) | 9 (1.6%) | 3679.4 / 3896.3 | 3.26 / 2.31 | 0.708 |
| ○ Stayed | 543 (97.8%) | 559 (98.4%) | – | – | – |
| **Overall Extractions** | 21 | 12 | 4262.3 / 4396.7 | 4.93 / 2.73 | 0.554 |

● = Implant placement (State 1); ○ = Implant prosthetic restoration, loaded (State 2); ✖ = Implant extraction (State 3); IR = Incidence Rate (per 1000 person-years); IRR = Incidence Rate Ratio

**Supplementary Table S3**. Kaplan–Meier survival and cumulative incidence for transition from State 2 to 3

| **Time (years)** | **Control Survival (%)** | **ECRR Survival (%)** | **Control Cumulative Incidence (%)** | **ECRR Cumulative Incidence (%)** | **Difference (ECRR − Control)** | **Number at Risk (Control / ECRR)** |
| --- | --- | --- | --- | --- | --- | --- |
| 1 | 99.6 | 99.8 | 0.4 | 0.2 | −0.2 | 545 / 561 |
| 3 | 99.2 | 99.7 | 0.8 | 0.3 | −0.4 | 492 / 515 |
| 5 | 99.2 | 99.0 | 0.8 | 1.0 | 0.2 | 406 / 433 |
| 10 | 96.2 | 97.8 | 3.8 | 2.2 | −1.7 | 47 / 57 |
|  |  |  |  |  |  |  |

6. Supplementary figures (captions and placeholders)


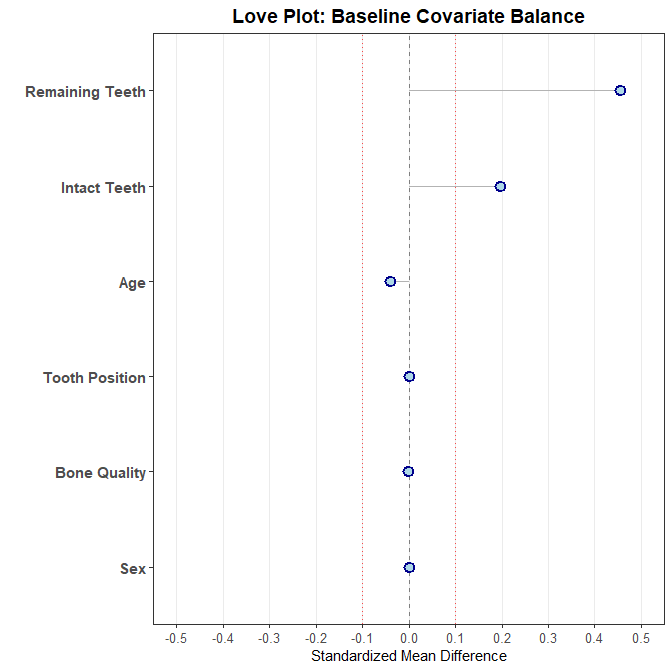


**Supplementary Figure S1**. Love plot showing baseline covariate balance (|SMD| ≤ 0.1 indicated by dotted lines).

**
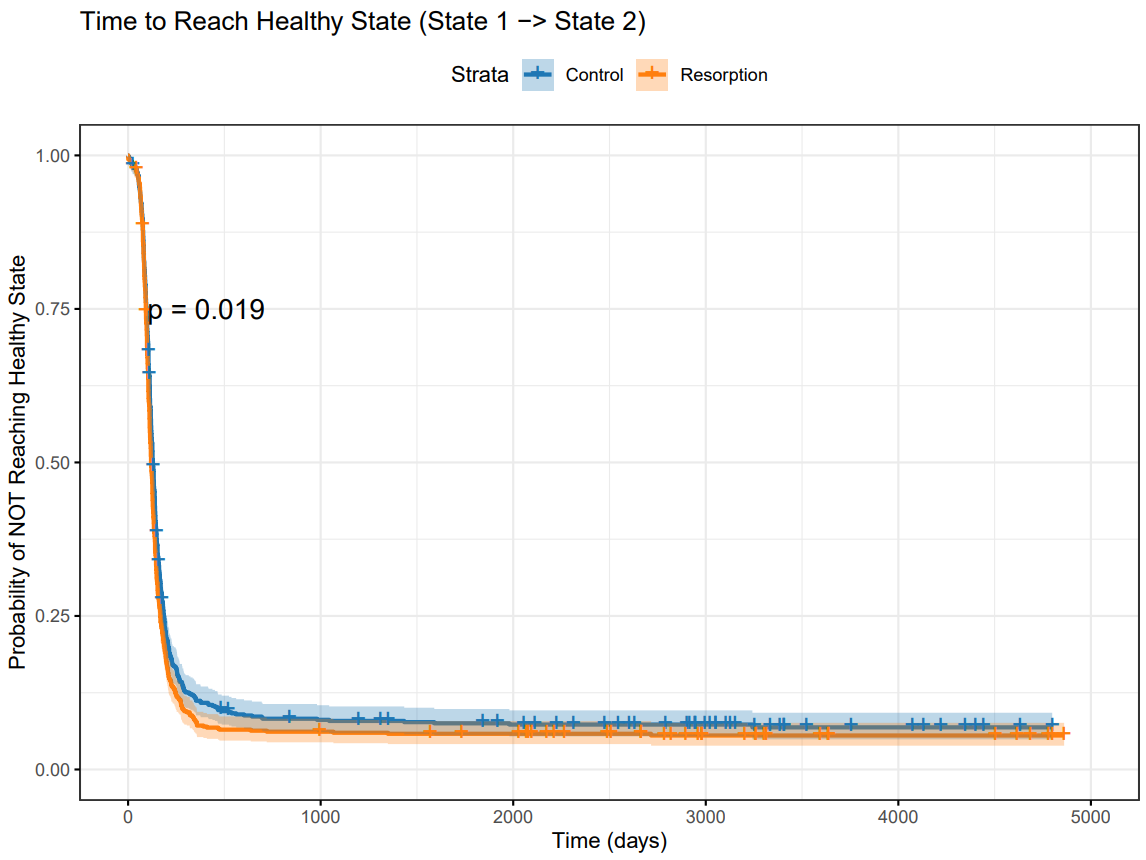
**

**Supplementary Figure S2**. Kaplan–Meier curves for time from implant placement (State 1) to healthy/loaded state (State 2) by group. Median times: 119 days (ECRR= Resorption) vs 129 days (control); log-rank χ² = 5.5, *p* = 0.02. Shaded areas indicate 95% confidence intervals; censored observations are marked with crosses.


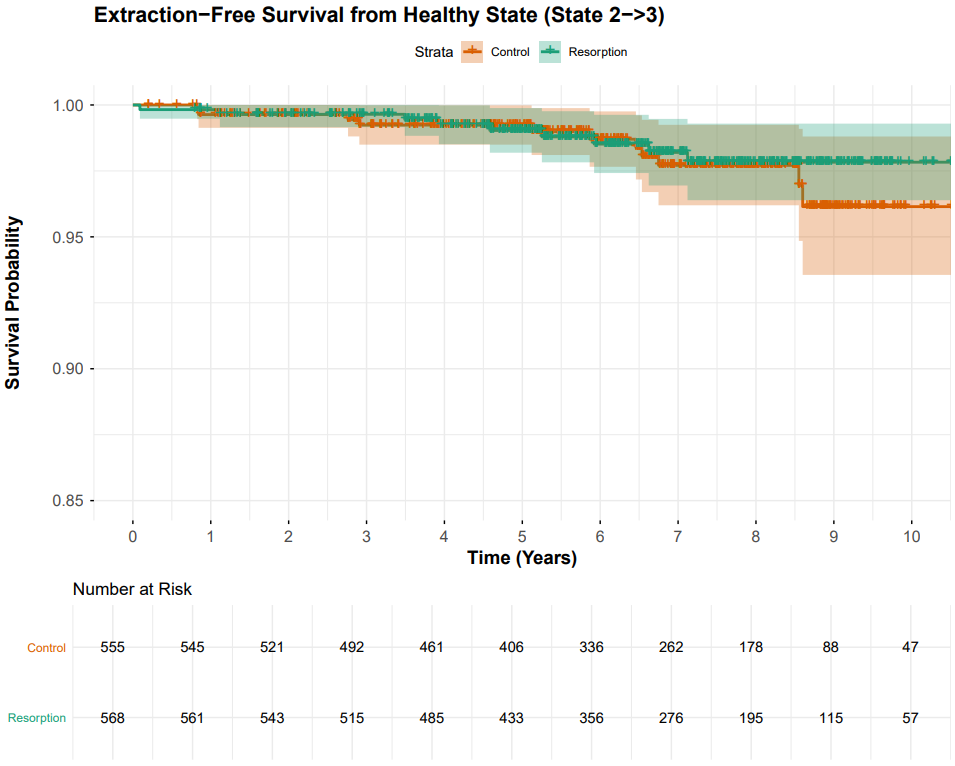


**Supplementary Figure S3**. Kaplan–Meier curves for time from healthy/loaded state (State 2) to implant extraction (State 3) by group; log-rank χ² = 0.004, *p* = 0.95. Shaded areas indicate 95% confidence intervals; censored observations are marked with crosses.ECRR= Resorption.

7. Software and reproducibility

Analyses were conducted in R (version 4.5.0) using *tidyverse*, *survival*, *survminer*, *cobalt*, *gee*, *lme4*, and *ggplot2* packages. Annotated code and scripts are available from the corresponding author on reasonable request to facilitate reproducibility.
